# Reversible Adaptive Plasticity: A Mechanism for Neuroblastoma Cell Heterogeneity and Chemo-Resistance

**DOI:** 10.3389/fonc.2012.00082

**Published:** 2012-08-02

**Authors:** Lina Chakrabarti, Thamara Abou-Antoun, Stanislav Vukmanovic, Anthony D. Sandler

**Affiliations:** ^1^The Joseph E. Robert Center for Surgical Care, Children’s National Medical CenterWashington, DC, USA; ^2^The Sheikh Zayed Institute for Pediatric Surgical Innovation, Children’s National Medical CenterWashington, DC, USA

**Keywords:** neuroblastoma, tumor cell plasticity, phenotypic switching, tumor heterogeneity, tumor cell adaptation

## Abstract

We describe a novel form of tumor cell plasticity characterized by reversible adaptive plasticity in murine and human neuroblastoma. Two cellular phenotypes were defined by their ability to exhibit adhered, anchorage dependent (AD) or sphere forming, anchorage independent (AI) growth. The tumor cells could transition back and forth between the two phenotypes and the transition was dependent on the culture conditions. Both cell phenotypes exhibited stem-like features such as expression of nestin, self-renewal capacity, and mesenchymal differentiation potential. The AI tumorspheres were found to be more resistant to chemotherapy and proliferated slower *in vitro* compared to the AD cells. Identification of specific molecular markers like MAP2, β-catenin, and PDGFRβ enabled us to characterize and observe both phenotypes in established mouse tumors. Irrespective of the phenotype originally implanted in mice, tumors grown *in vivo* show phenotypic heterogeneity in molecular marker signatures and are indistinguishable in growth or histologic appearance. Similar molecular marker heterogeneity was demonstrated in primary human tumor specimens. Chemotherapy or growth factor receptor inhibition slowed tumor growth in mice and promoted initial loss of AD or AI heterogeneity, respectively. Simultaneous targeting of both phenotypes led to further tumor growth delay with emergence of new unique phenotypes. Our results demonstrate that neuroblastoma cells are plastic, dynamic, and may optimize their ability to survive by changing their phenotype. Phenotypic switching appears to be an adaptive mechanism to unfavorable selection pressure and could explain the phenotypic and functional heterogeneity of neuroblastoma.

## Introduction

Despite extensive research, recurrent pediatric neuroblastoma remains an elusive disease with poor prognosis. Neuroblastoma, the most common extracranial solid tumor of childhood includes half of all neoplasms diagnosed in the first year of life (Gurney et al., 1997; Brodeur, 2003). It displays divergent behavior ranging from spontaneous regression to inevitable progression and death. Neuroblastoma is often responsive to standard treatments, resulting in tumor reduction, and a state of minimal residual disease (Matthay et al., 1999; De Bernardi et al., 2003). However, high-risk cases of neuroblastoma usually recur and are most often fatal (Tajiri et al., 2001; Woods et al., 2002; Maris, 2010; Yalcin et al., 2010; Jiang et al., 2011). The frequency of relapse and subsequent failure of treatment spurred the need to better understand the mechanism of resistance with the ultimate aim of designing more effective targeted therapies.

Tumor cell plasticity is the ability of a tumor cell to adapt to its environment and change its phenotype. This plasticity may allow tumor cells to reversibly turn on and off specific markers. It is described in several tumor types, including melanoma (Hoek et al., 2008) and glioblastoma (Bonavia et al., 2011). Tumor cell adaptation is an important phenomenon as it is believed to constitute a major mechanism enabling tumors to evade surveillance of the immune system, survive unfavorable conditions, or escape radio- or chemotherapy. The human neuroblastoma cell line SK-N-SH revealed phenotypic plasticity featuring morphological and biochemical inter-conversion in culture (Ross et al., 1983). The cell lines SH-SY-5Y and SK-N-MC demonstrated adaptation to hypoxia in which the neuroblastoma cells transition from an adherent phenotype to a highly migratory and invasive cell type (Poomthavorn et al., 2009). The dynamic behavior of tumor cells has been shown in other tumor models like glioblastoma (Jiang et al., 2011) and melanoma (Roesch et al., 2010). The major mechanism considered for tumor adaptation is genetic instability followed by the selection of resulting phenotypes that adapt best to the environmental conditions. This represents a unidirectional path resulting in adapted tumor cell phenotypes that could be characterized by increased aggressiveness, metastatic potential, or resistance to therapeutic interventions. In this manner, tumor development can be regarded as a process of Darwinian evolution (Marusyk and Polyak, 2010). The cellular source of this evolution could be rare cancer stem cells giving rise to tumor cells with high-risk characteristics (Lonardo et al., 2010; O’Brien et al., 2010). Although the existence of a minor subset of cancer cells that have the unique ability to self-renew has been demonstrated by multiple research groups, the concept of cancer stem cells remains a subject of debate.

Another mechanism that could afford tumors aggressive, resistant behavior, and heterogeneity is an epithelial to mesenchymal transition (EMT; Creighton et al., 2009). In this process, epithelial cells detach and acquire mesenchymal characteristics facilitating cell migration. Once migrated, these cells can either remain mesenchymal or de-differentiate into epithelial cells by a process known as mesenchymal to epithelial transition (MET; Thiery and Sleeman, 2006; Creighton et al., 2010). These findings imply that tumor cell plasticity can be achieved by cellular adaptation at the single cell level, rather than through selection of particular tumor variants. The findings of invasive and proliferative gene expression signatures of melanoma growing *in vivo* (Hoek et al., 2008) suggests that other forms of tumor cell transitions exist that need not be based on (de)differentiation.

We describe a novel form of tumor cell plasticity characterized by reversible adaptive phenotypic transformation. Two defined neuroblastoma phenotypes both having stem-like characteristics were induced using distinct culture conditions. Identification of specific molecular markers enabled us to observe both phenotypes in tumors growing *in vivo*, irrespective of the phenotype originally inoculated in mice. This finding explains tumor heterogeneity and establishes that reversible or adaptive phenotypic conversion occurs *in vivo*. Facing chemotherapeutic intervention, tumors *in vivo* temporarily showed preference for the chemo-resistant phenotype. Simultaneous targeting of both tumor cell phenotypes in a mouse model of neuroblastoma resulted in delay in tumor growth and emergence of new phenotypes. These observations describe adaptive phenotypic transformation as a mechanism of tumor cell plasticity that enables tumors to escape unfavorable environmental conditions.

## Materials and Methods

### Animals

Female A/J mice (6 weeks old) were purchased from Jackson Laboratory (Bar Harbor, ME, USA). The animals were acclimated for 4–5 days prior to tumor challenge. All procedures were approved by Institutional Animal Care and Use Committee.

### Murine and human cell lines

Neuro2a is the murine neuroblastoma cell line derived from AJ mice. IMR-32 and SK-N-SH are human cell lines derived from MYCN amplified and non-amplified neuroblastoma tumors, respectively. All cell lines were purchased from ATCC (Manassas, VA, USA). Neuro2a and SK-N-SH cells were cultured in DMEM (Gibco, Carlsbad, CA, USA) while IMR-32 cells were cultured in EMEM (Lonza, Walkersville, MD, USA); both media containing 10% fetal bovine serum (FBS, Gibco), 0.5% penicillin/streptomycin, and 10% l-glutamine. The cells grew as anchorage dependent (AD) cells. Anchorage independent (AI) tumorspheres from each cell line were grown in NeuroCult complete media consisting of NeuroCult Neural Stem Cell (NSC) Basal medium, 1/10 NeuroCult NSC Proliferation supplements, 20 ng/ml EGF, 10 ng/ml bFGF, and 2 μg/ml Heparin. NeuroCult media, supplements, and growth factors were all purchased from Stem Cell Technologies (Vancouver, BC, Canada).

### Cell cycle analysis

The AD and AI cells were harvested from culture medium, washed in cold PBS, and fixed in 70% ethanol at −20°C for 2 h. Cells were centrifuged, washed with cold PBS, and resuspended in propidium iodide mix (40 μg/ml PI and 0.1 mg/ml RNAse in PBS). After 30 min incubation at 37°C cells were analyzed in FACSCalibur (Becton Dickinson, San Jose, CA, USA).

### MTT assay

Metabolic activity of the proliferating cells after Doxorubicin (100 ng/ml) treatment was determined by the colorimetric absorbance of the MTT assay at 24, 48, and 72 h post treatment. Briefly, control and treated AD and AI cells (5000 cells/well) were plated in 96-well plates and MTT [3-(4,5-dimethylthiazolyl-2)-2,5-diphenyltetrazolium bromide] (Sigma, St. Louis, MO, USA) was added to each well at a concentration of 10 mg/ml. After 4 h, the purple formazan formed from the reduction of MTT was dissolved in DMSO and the absorbance was measured at 540 nm in a plate reader.

### Apoptosis assay

Control and irradiated AD and AI cells were analyzed for apoptosis by flow cytometry. Briefly, cells were washed with 1× Annexin binding buffer and stained with APC-conjugated AnnexinV for 20 min followed by 7-AAD for 5 min. Cells were analyzed immediately for AnnexinV/7-AAD expression using FACSCalibur.

### Differentiation assay

Mouse and human AD and AI cells were compared for their multi-lineage differentiation potential following placement in poly-d-lysine coated coverslips with differentiation media for 10–14 days. Fixed cells were stained with markers of stem cells (nestin), neurons (βIII-tubulin, Tuj1), oligodendrocytes (O4), and astrocytes (glial fibrillary acidic protein, GFAP) as described in *Immunofluorescence assay*.

### *In vivo* tumor growth and drug treatments

Mice were injected subcutaneously with 1 × 10^6^ AD or AI Neuro2a cells and divided into four groups; AD-treated, AI-treated, AD-untreated, and AI-untreated. For the chemotherapeutic treatment regimen, doxorubicin (2.5 mg/kg) was administered twice a week intraperitonealy (i.p.) and metformin daily in drinking water to the treated groups starting on post-inoculation day 1. For inhibiting the growth factor receptors, a combined dose of erlotinib (EGFR inhibitor, 50 mg/kg) and PD173074 (FGFR inhibitor, 50 mg/kg) was administered i.p. every alternate day to the treated groups starting on post-inoculation day 1. To treat the mice simultaneously with chemotherapy and growth factor inhibition, doxorubicin, erlotinib, and PD173074 were administered twice a week and metformin was given daily in drinking water starting on post-inoculation day 1. Tumor growth was monitored on alternate days. Tumors reaching 5, 10, or 15 mm in diameter were resected. Tumors were either fixed in 4% paraformaldehyde (PFA) and frozen for immunofluorescence staining or fixed in formalin and processed for immunohistology or digested with collagenase/dispase/DNAse for flow cytometric analysis. The digested tumor was also used to extract protein for Western blot analysis.

### Immunofluorescence assay

Ten micron frozen sections of 10 and 15 mm diameter size tumors or the cultured cells on coverslips were subjected to inmmunofluorescence assay using the antibodies: rabbit anti-nestin (1:500, Abcam, Cambridge, MA, USA), doublecortin (Dcx, 1:500, Abcam), neural cell adhesion molecule (NCAM, 1:1000, Cell Signaling, Danvers, MA, USA), β-catenin (1:1000, Cell Signaling), survivin (1:1000, Cell Signaling), and platelet derived growth factor receptor beta (PDGFRβ, 1:100, Santa Cruz Biotechnology, Santa Cruz, CA, USA), mouse anti-Tuj1 (1:500, Covance, Princeton, NJ, USA), O4 (1:1000, Stem Cell Technologies), GFAP (1:1000, Sigma), and microtubule associated protein-2 (MAP2, 1:1000, Sigma). The secondary antibodies used were AlexaFluor 488, AlexaFluor 546, and AlexaFluor 633 conjugated goat anti-rabbit or anti-mouse IgG (1:200, Invitrogen, Carlsbad, CA, USA). All fluorescent images were taken on aLSM confocal microscope (Carl Zeiss Inc., Germany) using 20× or 40× objectives.

### Tumor histology

The formalin fixed tumors grown from AD or AI Neuro2a cells were sectioned by a pathologist and H&E staining was performed to examine for histopathologic differences between the tumors derived from the two phenotypes of the Neuro2a cells.

### Western blot analysis

Protein concentration was determined according to manufacturer’s instruction using BCA Protein Assay Kit (Pierce, Rockford, IL, USA). Twenty micrograms of protein homogenate were loaded per well for electrophoresis after which the proteins were transferred to polyvinylidene difluoride membranes and blocked with 5% milk. The blots were incubated overnight with a 1:1000 dilution of rabbit anti-nestin, Dcx, NCAM, β-catenin, survivin, PDGFRβ, Telomerase, Gli1 (Santa Cruz biotechnology), vascular endothelial growth factor (VEGF, Santa Cruz Biotechnology), and mouse anti-MAP2 and VEGFR2 (Santa Cruz Biotechnology) followed by HRP-conjugated anti-rabbit or anti-mouse secondary antibodies (1:2000, Pierce). Blots were developed by chemiluminescence using SuperSignal Kit (Pierce). Rabbit anti-GAPDH (Cell Signaling) was used as a control for protein loading variations.

### Flow cytometric phenotyping

The digested tumor or the cultured AD and AI cells were phenotyped according to the slightly modified protocols of Leung et al. (1992) and Daniel et al. (2008). Briefly, the cells were centrifuged at low speed, fixed in 4% PFA, washed with PBS containing 2% FBS (FACS buffer), and resuspended in 100 μl FACS buffer containing 0.03% TritonX-100. The cells were incubated with primary antibodies (nestin, MAP2, β-catenin, and PDGFRβ) for 30 min at room temperature. No TritonX-100 was added for PDGFRβ staining. The cells were washed with FACS buffer and incubated with FITC-conjugated goat anti-rabbit and APC-conjugated goat anti-mouse secondary antibodies for 20 min. After additional washes, the cells were analyzed in FACSCalibur. Tumor cells were gated from forward scatter/side scatter plots.

### Statistical analysis

Data are presented as mean ± S.D. Two-tailed Student’s *t*-test was used to determine statistical significance between groups. A probability level of *p* < 0.05 was considered to be statistically significant.

## Results

### Reversible phenotypic adaptation in neuroblastoma

**Figure 1:**
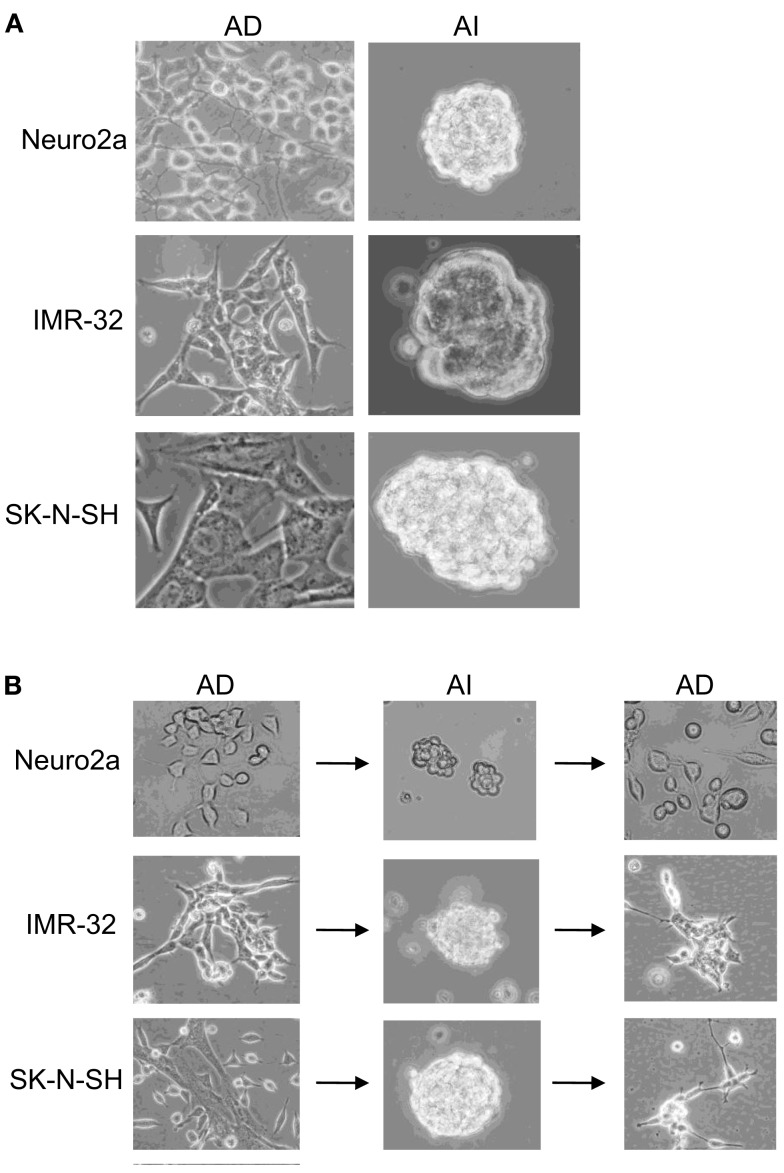
**Reversible adaptive plasticity of neuroblastoma cell lines. (A)** Neuro2a, IMR-32, and SK-N-SH cells cultured in DMEM + 10% FBS (D10) or Neurocult complete (NC) medium formed a monolayer of anchorage dependent (AD) adhered cells or anchorage independent (AI) tumorspheres, respectively. **(B)** Dissociated AI cells, originally derived from AD cells readily reversed their phenotype and formed AD monolayers when cultured in the D10 medium. Images acquired with 20× objective.

Mouse (Neuro2a) and human (IMR-32 and SK-N-SH) cell lines, cultured in D10 media exhibited an AD phenotype characterized by growth in a monolayer. Transfer to serum-free NeuroCult complete (NC) media supplemented with EGF and FGF induced an AI phenotype, characterized by detachment of cells and formation of floating tumorspheres (Figure 1A). AI cells from all the cell lines readily reformed tumorspheres when cultured in NC media, but adhered and formed monolayers when cultured in the D10 media (Figure 1B). Thus, neuroblastoma cultures facing distinct growth promoting conditions can transition to and from two distinct phenotypes which are defined by tumor cell adherence characteristics. We have observed a similar phenomenon of adaptive growth patterns in melanoma and rhabdomyosarcoma cell lines (Unpublished data).

**Figure 2:**
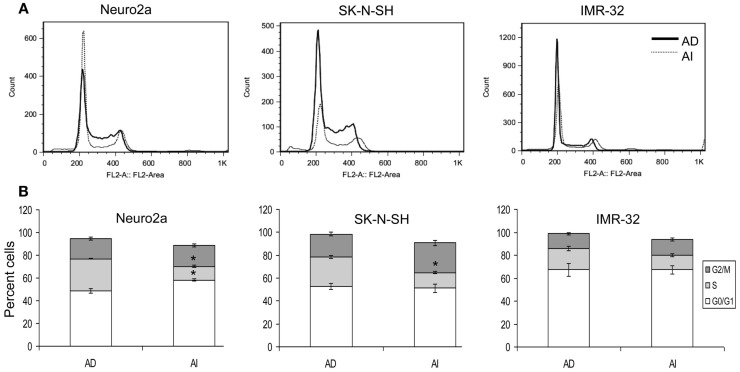
**Cell cycle analysis of AD and AI phenotypes of neuroblastoma cell lines**. **(A)** Cell cycle analysis of Neuro2a, SK-N-SH, and IMR-32 cells using propidium iodide. **(B)** Graphical representation of the cell cycle analysis reveals that AI phenotype of Neuro2a and SK-N-SH cells has significantly fewer numbers of cells in S-phase compared to their AD phenotype. Data points expressed as mean ± S.D. (*n* = 3). *p < 0.01 by Student’s *t*-test.

**Figure 3:**
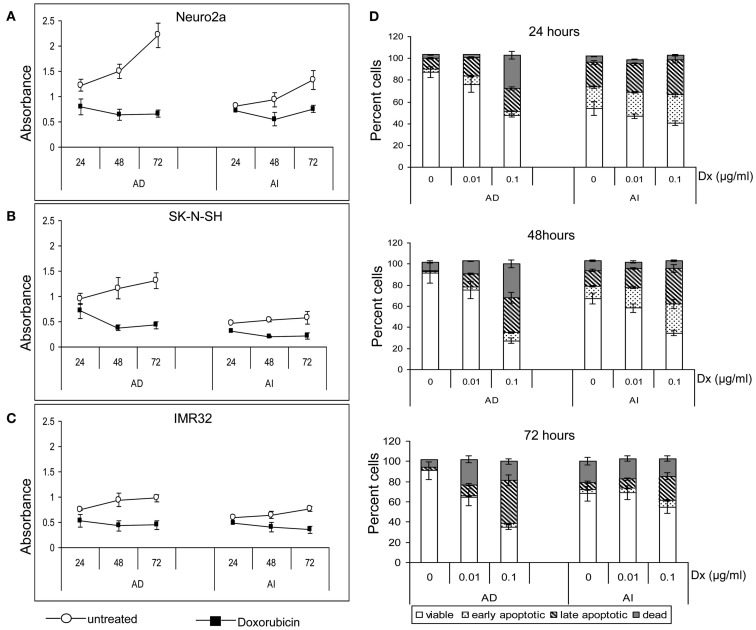
**Effect of doxorubicin on metabolic activity and apoptosis of neuroblastoma cell lines**. MTT assay of the untreated and doxorubicin treated Neuro2a **(A)**, SK-N-SH **(B)**, and IMR-32 **(C)** cells of either AD or AI phenotype revealed slower metabolic activity of the AI cells. Doxorubicin lowered the metabolic activity of both the phenotypes of all cell lines. **(D)** Graphical representation of the apoptosis assay at 24, 48, and 72 h following doxorubicin (Dx; 0, 0.01, and 0.1 μg/ml) treatment shows that the AI cells are more resistant to doxorubicin than the AD cells. The change in viability between the treated and untreated groups of AI cells is less for all time points and drug doses. Data points expressed as mean ± S.D. (*n* = 3).

Anchorage independent phenotypes grew slower than AD phenotypes in all cell lines as indicated by cell cycle analysis and MTT assay (Figures 2A,B and 3A–C). The AD cells were also found to be more sensitive to the chemotherapeutic agent doxorubicin than the AI cells (Figure 3D).

### Stem cell-like qualities are present in both AD and AI phenotypes

**Figure 4:**
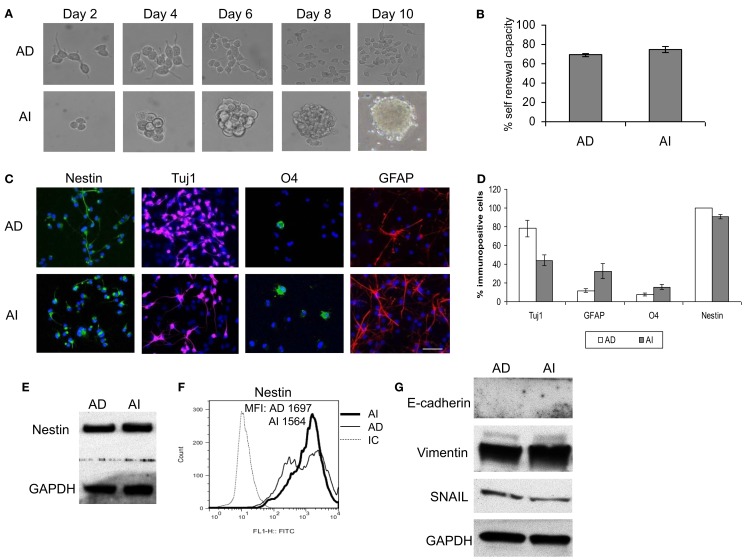
**Stem cell-like properties of AD and AI phenotypes of mouse neuroblastoma cell line. (A)** Dissociated AI or AD phenotypes of Neuro2a cells reseeded at limited dilution in NC or D10 media exhibited self-renewal capability over time by reforming tumorspheres or growing as adhered cells, respectively. **(B)** Graphical representation of the percent self-renewal capacity of the AD and AI cells as measured by the percent of wells (seeded at 1 cell/well) growing adherent cells or tumorspheres, respectively. **(C)** Neuro2a cell-derived AI tumorspheres and AD adhered cells were allowed to differentiate followed by immunostaining for stem cells (nestin), neurons (Tuj1), oligodendrocytes (O4), and GFAP (astrocytes). **(D)** Graphical representation of the percent immunopositive cells revealed multipotency and ubiquitous expression of nestin for both phenotypes. Data points expressed as mean ± S.D. (*n* = 3). **(E)** Western blot analysis supports the abundant expression of nestin by both AD and AI phenotypes of Neuro2a cells. **(F)** Flow cytometric analysis supports that nestin is a ubiquitous marker of both tumor cell phenotypes. MFI, mean fluorescence intensity. **(G)** Western blot analysis showing complete absence of E-cadherin and abundance of vimentin and SNAIL proteins in both AD and AI phenotypes of neuro2a cells. Scale bar, 100 μm.

Cancer stem cell properties include self-renewal capacity, potent *in vivo* tumorigenic activity, and an undifferentiated state with the ability to differentiate (Al-Hajj et al., 2003; Singh et al., 2003; Dean et al., 2005; Bao et al., 2006). The phenotypic transition could be a property of a rare cell subpopulation within the cell lines (such as cancer stem cells) or a general cell property. To evaluate these possibilities we performed a self-renewal assay that is considered a stem-like quality. AI and AD forms of Neuro2a cells were dissociated and reseeded at a limiting dilution. Cells cultured in NC media reformed tumorspheres, while those cultured in D10 formed monolayers of adhered cells (Figure 4A). The efficiency of clonal growth of cells seeded at 1 cell/well was similar in AI and AD phenotypes, suggesting self-renewal potential in both phenotypes (Figure 4B).

**Figure 5:**
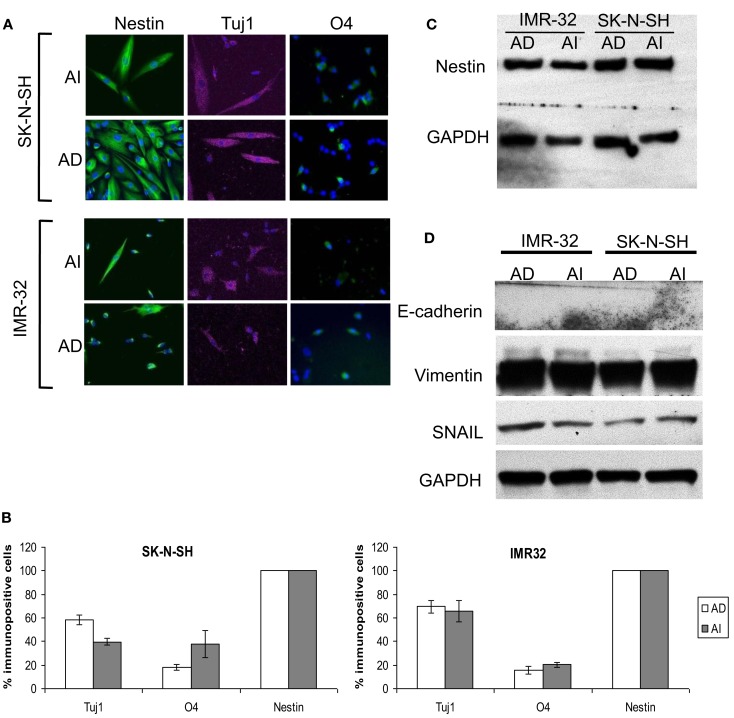
**Stem cell-like properties of AD and AI phenotypes of human neuroblastoma cell lines**. **(A)** SK-N-SH and IMR-32 cell-derived AI tumorspheres and AD adhered cells were allowed to differentiate followed by immunostaining for stem cells (nestin), neurons (Tuj1), oligodendrocytes (O4), and GFAP (astrocytes). Both the phenotypes of each cell type differentiated into neurons and oligodendrocytes but no astrocyte was detected. **(B)** Graphical representation of the percent immunopositive cells revealed multipotency and abundant expression of nestin for both the phenotypes. Data points expressed as mean ± S.D. (*n* = 3). **(C)** Western blot analysis supports the abundant expression of nestin by both AD and AI phenotypes. **(D)** Western blot analysis showing absence of E-cadherin and abundance of vimentin and SNAIL proteins in both AD and AI phenotypes of human neuroblastoma cell lines.

We also assessed the differentiation potential of AI and AD phenotypes as another characteristic of stem cells. Staining with neuronal (Tuj1), oligodendrocyte (O4), and astrocyte (GFAP) markers revealed differentiation into all three lineages for both phenotypes of Neuro2a (Figures 4C,D). The human cell lines differentiated readily into neurons and oligodendrocytes, but not into astrocytes (Figure 5A). Immunoflourescence, Western blot, and flow cytometric analysis indicate that the stem cell marker, nestin is expressed in both AD and AI phenotypes (Figures 4D–F and 5B,C). Furthermore, expression of other suggested stem cell markers (Sox2, CD133, and CD44) were not different between the AI and AD phenotypes (data not shown). Taken together, neither phenotype appears specifically enriched for putative cancer stem cells, but both have stem-like qualities. In addition, the phenotypic conversion does not alter stem-like characteristics of Neuro2a cells.

### The Neuro2a phenotypic adaptation is distinct from epithelial to mesenchymal transition

Loss of E-cadherin and upregulation of β-catenin, vimentin, and SNAIL are classic characteristics for EMT. E-cadherin was not detected in either AI or AD phenotype while vimentin and SNAIL were equally abundant in both (Figures 4G and 5D). Thus, besides the fact that neuroblastoma is not an epithelial tumor, the reversible phenotypic conversion described is inconsistent with the molecular changes observed in either EMT or MET.

### The two neuroblastoma phenotypes express distinct molecular signatures *in vitro*

**Figure 6:**
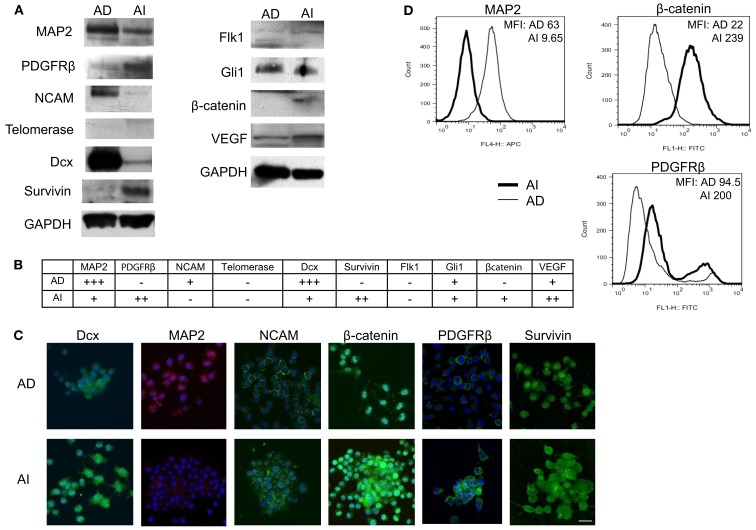
**Molecular markers differentiate AD and AI phenotypes of neuroblastoma *in vitro***. **(A)** Western blot analysis of proteins that play vital role in neuroblastoma homeostasis revealed differences in expression of PDGFRβ, MAP2, Dcx, NCAM, survivin, and β-catenin between the AD and AI phenotypes. **(B)** The table represents relative protein expression of AD and AI cell types as determined by Western blot analysis. **(C)** Immunofluoresence analysis *in vitro* demonstrated that MAP2 is exclusively expressed by the AD cells whereas β-catenin and PDGFR-β is overexpressed in AI cells compared to the AD cells. **(D)** Flow cytometric analysis confirmed that MAP2 is expressed by the AD cells only, whereas β-catenin and PDGFRβ are overexpressed in AI cells. Scale bar, 50 μm. MFI, mean fluorescence intensity.

To determine whether the AI and AD phenotypes of Neuro2a cells have distinct molecular markers, we analyzed expression of eleven proteins that play a vital role in neuroblastoma homeostasis. These included proteins regulating proliferation (β-catenin/FZD1, SHH/Gli1/PTCH1, TrkB/BDNF, PDGFR), apoptosis (FZD1, Gli1), chemo-resistance (FZD1, Telomerase), angiogenesis (VEGF/VEGFR, PDGFRβ, SHH), metastasis (SHH, MAP2, TrkB), adhesion and migration (NCAM, TrkB, Dcx), and differentiation and neurite extension (MAP2, Dcx) (Eggert et al., 2000; Oltra et al., 2005; Nakamura et al., 2006; Flahaut et al., 2009; Korja et al., 2009; Krishnan et al., 2009; Bahnassy et al., 2010; Oue et al., 2010; Souzaki et al., 2010; Wesbuer et al., 2010; Zhang et al., 2010; Schiapparelli et al., 2011). Western blot analysis revealed differences in expression of PDGFRβ, MAP2, Dcx, NCAM, survivin, and β-catenin (Figures 6A,B), thereby identifying these molecules as potential unique markers of AI or AD phenotypes. To test the value of these molecules as markers of *in vivo* cellular commitment to AD or AI phenotypes we performed immunofluorescence microscopy. This analysis confirmed the findings for MAP2, β-catenin, and PDGFRβ, while staining for Dcx, NCAM, and survivin did not discriminate (Figure 6C). Flow cytometric analysis confirmed differences in the expression of MAP2, β-catenin, and PDGFRβ while all neuroblastoma cells were nestin positive (Figure 6D). These results showed that Nestin + MAP2+ cells are indicative of the AD phenotype while Nestin + MAP2− are markers of the AI phenotype.

### Tumor cell heterogeneity *in vivo*

**Figure 7:**
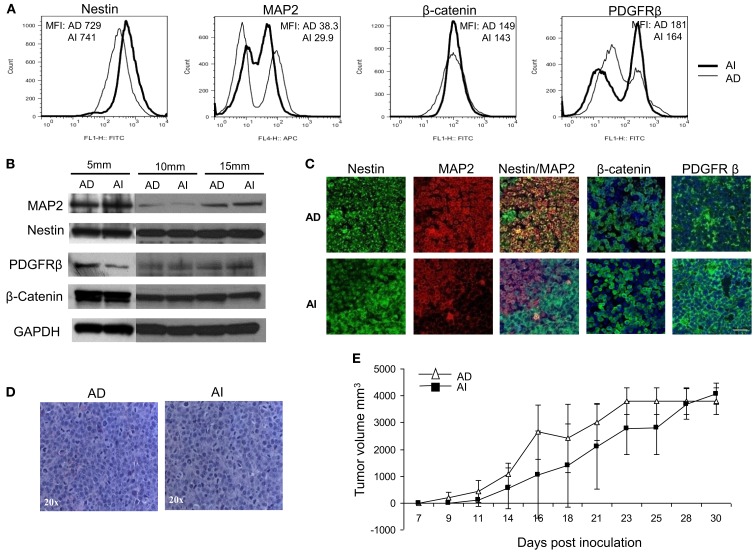
**Neuroblastoma tumor cell heterogeneity in mouse model. (A)** Flow cytometric phenotyping analysis on mouse neuroblastoma tumors of 10 mm diameter showed no remarkable difference in the expression of MAP2, β-catenin, and PDGFRβ in the two forms of tumor. MFI, mean fluorescence intensity. **(B)** Western blot analysis on mouse neuroblastoma tumors (5, 10, and 15 mm in diameters) revealed the heterogeneity of MAP2, PDGFRβ, and β-catenin expression in either forms of tumor suggesting that phenotypic transitions occur *in vivo*. **(C)** Immunofluorescence staining on frozen sections from mouse neuroblastoma tumors (grown from either AD or AI cells) of 10 mm diameter supports the flow cytometric and Western blot analysis of tumor cell heterogeneity. **(D)** H&E staining of tumors reveals that both AD and AI forms of Neuro2a cells gave rise to histopathologically similar tumors in mice. **(E)** *In vivo* tumorigenic potential of the AD and AI phenotypes of Neuro2a cells were compared by inoculating the mice with either form of cell phenotype and measuring the tumor over time. Both cell types gave rise to very large tumors and the growth rates were indistinguishable. Data points expressed as mean ± S.D. (*n* = 12). Scale bar, 50 μm.

To determine the *in vivo* representation and stability of the *in vitro* phenotypes, mice were inoculated separately with Neuro2a cells of AD or AI phenotypes. Nestin, MAP2, β-catenin, and PDGFRβ expression were analyzed in tumors reaching 5, 10, and 15 mm in diameter. Flow cytometry and Western blot analyses revealed similar abundance of each protein in tumors of corresponding sizes irrespective of the phenotype injected (Figures 7A,B). This finding suggests that either both AI and AD phenotypes were present *in vivo*, or that a third phenotype containing both AI and AD markers was predominant *in vivo*. To distinguish between these possibilities we stained frozen tumor sections with nestin, MAP2, PDGFRβ, and β-catenin specific antibodies. This analysis showed heterogeneity of cells *in situ* (Figure 7C). In light of ubiquitous nestin expression, scattered areas with differential MAP2, PDGFRβ, and β-catenin staining represent Neuro2a cells of AD (MAP2) or AI (PDGFRβ and β-catenin) phenotype. This conclusion is also supported by co-staining of nestin and MAP2 showing both Nestin + MAP2+ and Nestin + MAP2− cells in tumors irrespective of their phenotype prior to inoculation (Figure 7C). The pockets of cells with distinct phenotypes were not localized to a specific region in the tumor mass, but seemed randomly distributed (data not shown). Not surprisingly, the histopathologic appearance (Figure 7D) and *in vivo* growth (Figure 7E) of the tumors were also indistinguishable. Therefore, Neuro2a cells driven to AI or AD phenotype *in vitro* display a tendency to transition, establish, and maintain an approximate 1:1 ratio of the two phenotypes *in vivo*.

**Figure 8:**
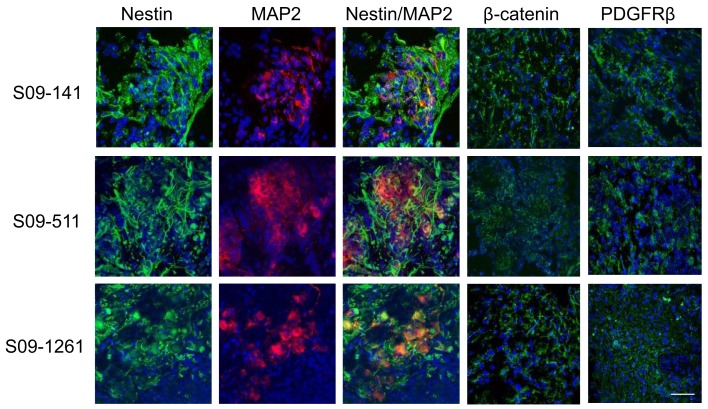
**Cell heterogeneity in human primary neuroblastoma tumors**. Immunofluorescence staining with MAP2, β-catenin, and PDGFRβ on frozen sections of three human primary neuroblastoma specimens revealed scattered areas with differential MAP2, PDGFRβ, and β-catenin staining indicating similar heterogeneity of cells as seen in mouse tumors. Scale bar, 50 μm.

The coexistence of AI and AD phenotypes *in vivo* could be unique to Neuro2a cells or cell lines in general, and may not be a reflection of events that occur in primary neuroblastoma tumors. To address this issue, frozen sections of three human neuroblastoma specimens were co-stained with nestin and MAP2 and separately with PDGFRβ and β-catenin specific antibodies. This analysis revealed similar heterogeneity of cells as seen in mouse tumors indicating that phenotypic heterogeneity exists in primary human neuroblastomas (Figure 8).

### Chemotherapy alters the heterogeneity of the tumor cells *in vivo*

**Figure 9:**
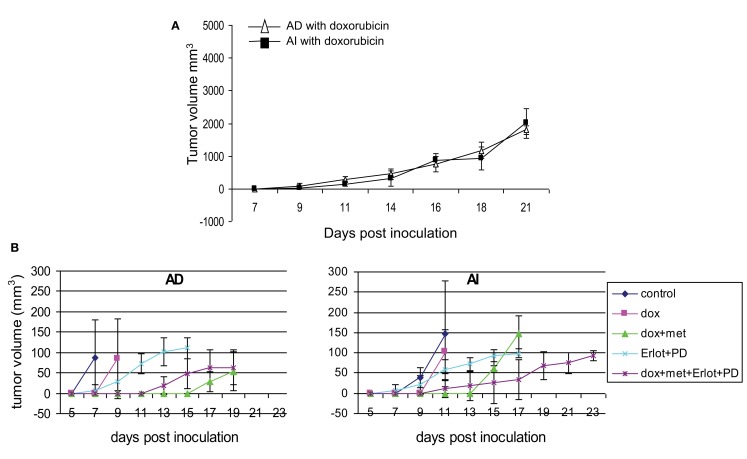
**Effect of chemotherapy on tumor growth**. **(A)** Doxorubicin treatment slowed down tumor growth in mice inoculated with AD) and AI forms of Neuro2a cells. Data points expressed as mean ± S.D. (*n* = 5). **(B)** Combined treatment with doxorubicin, metformin, Erlotinib (EGFR blocker), and PD173074 (FGFR blocker) delayed the initial onset of tumor in mice challenged with AD or AI phenotypes of Neuro2a. Data points expressed as mean ± S.D. (*n* = 6). These tumors were harvested at a size of 5 mm diameter.

**Figure 10:**
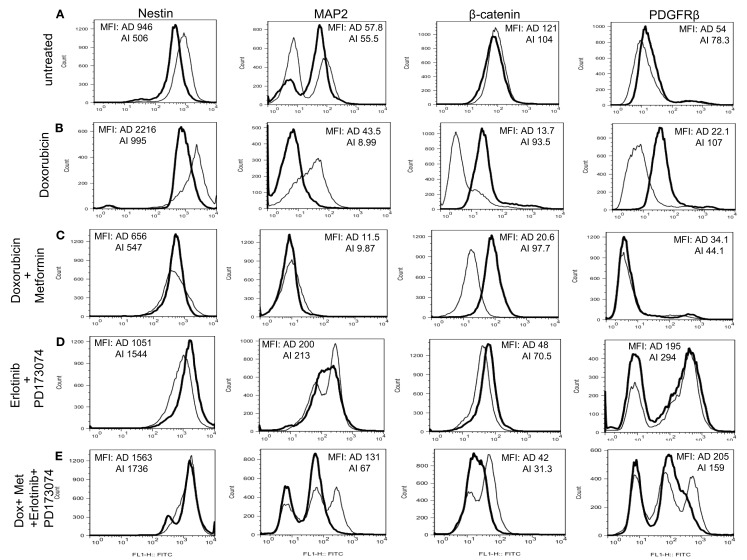
**Chemotherapy alters heterogeneity of cells in small tumors**. **(A)** Flow cytometric analysis on mouse neuroblastoma tumors of 5 mm diameter showed no remarkable difference in the expression of MAP2, β-catenin, and PDGFRβ in the two forms of tumor (grown from either AD or AI cells). **(B)** Doxorubicin treatment had no effect on the cellular heterogeneity on tumors of 5 mm diameter grown from AD cells; whereas the AI-tumors of similar size lost their heterogeneity, remained in their native form, and did not transition during doxorubicin treatment. **(C)** Combination of doxorubicin and metformin prevented the AD phenotype from establishing itself in the early stage (5 mm diameter) of tumor formation as defined by the absence of MAP2+ cells and the cells remained in the AI form. **(D)** Combined dose of Erlotinib (EGFR inhibitor) and PD173074 (FGFR inhibitor) prevented the AI phenotype from establishing itself in the early stage (5 mm diameter) of tumor formation as defined by the presence of MAP2+ cells and the cells remained in the AD form. **(E)** Combination of chemotherapy and growth factor receptor inhibitors resulted in the emergence of newer phenotypes as evident from the presence of unique/unfamiliar populations of MAP2+ and PDGFRβ+ cells. MFI, mean fluorescence intensity. AD, AI.

**Figure 11:**
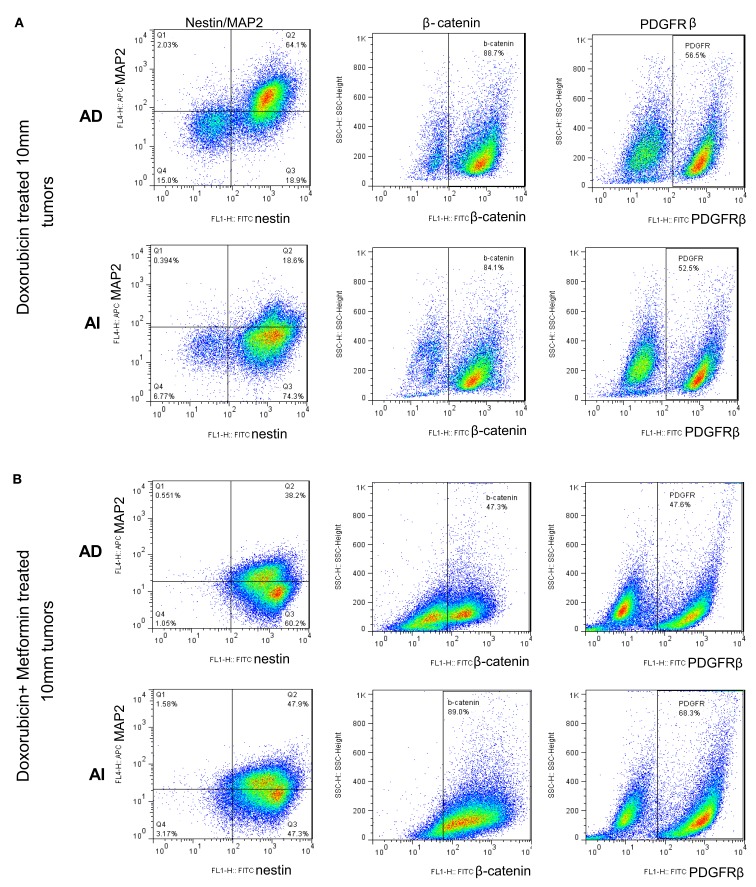
**Large doxorubicin and doxorubicin/metformin double-resistant tumors resume phenotypic heterogeneity**. Flow cytometric analysis on large (10 mm diameter) mouse neuroblastoma tumors treated with doxorubicin **(A)** and doxorubicin/metformin **(B)** showed no remarkable difference in the expression of MAP2, β-catenin, and PDGFRβ in the two forms of tumor (grown from either AD or AI cells). Apparently either treatment displayed both AD (MAP2+) and AI (MAP2−) phenotypes.

Since Neuro2A AI and AD phenotypes were differentially susceptible to doxorubicin exposure *in vitro*, we sought to determine the effects, if any, doxorubicin might exert on the Neuro2a phenotype of cells growing *in vivo*. Mice inoculated with AD and AI forms of Neuro2a cells were treated with doxorubicin. Initial tumor growth was found to be slower in the doxorubicin treated mice compared to the untreated mice (Figures 9A,B). At an early stage (5 mm in diameter), doxorubicin treated AD tumors maintained their heterogeneity similar to the untreated tumor (Figure 10A); whereas the AI-tumors remained in AI form and did not transition (Figure 10B). This observation shows that doxorubicin initially prevented transition to the more sensitive AD phenotype. Interestingly, as the tumors grew larger (10–15 mm in diameter), they became resistant to doxorubicin, displayed faster growth *in vivo* (Figure 9A), and both AD and AI forms were reconstituted (Figure 11A).

Metformin added to doxorubicin prevents relapse in xenografts induced by diverse cancer cell types including breast, prostate, and lung cancer cell lines (Iliopoulos et al., 2011). We therefore extended our study to address the effects of combination therapy (doxorubicin plus metformin) on tumor cell heterogeneity. The combined effects were remarkably more suppressive on tumor growth (Figure 9B) and also concurrently prevented the AD phenotype from establishing itself in early stage tumor formation as defined by the absence of MAP2+ cells irrespective of AD or AI inoculation (Figure 10C). The cells remained in the AI form while they adapted to the slow-growing, resistant phenotype (Figure 11B). These results show that doxorubicin-based chemotherapeutic treatment is preferentially effective on neuroblastoma cells of one phenotype and that in response to treatment, tumors temporarily transition into the more resistant phenotype. After chemo-resistance is established and tumors rebound, the heterogeneity of the phenotypes is re-established.

### Growth factor receptor inhibition targets the AI phenotype of the tumor cells *in vivo*

As the chemotherapeutic agents tested targeted the AD phenotype, we postulated that the AI phenotype could be targeted *in vivo* by inhibiting the growth factors used to induce this phenotype *in vitro*. Small molecule inhibitors of EGF (Erlotinib) and FGF (PD173074) receptors in combination prevented the growth of the AI phenotype in small tumors (5 mm) and resulted in tumors primarily containing the AD phenotype (Nestin + MAP2+) irrespective of the AD or AI phenotype inoculated (Figure 10D).

### Emergence of new phenotypes following chemotherapy combined with growth factor receptor inhibition

The collective findings showed that targeting either phenotype drove the cells into the other type, suggested that simultaneously targeting both phenotypes could prevent the reversible phenotypic adaptation from occurring. To test this hypothesis mice were treated with doxorubicin (twice a week), metformin (daily), Erlotinib (twice a week), and PD173074 (twice a week) starting a day after their tumor challenge with AD or AI phenotypes of Neuro2a cells. This combined therapy, significantly delayed the initial onset of tumor growth for both phenotypes (Figure 9B), but resulted in the emergence of newer phenotypes as evident from the presence of unique/unfamiliar populations of MAP2+ and PDGFRβ+ cells (Figure 10E). Taken together these results suggest that neuroblastoma tumor cells use their plastic adaptive phenotypic transformation as a tool to survive unfavorable selection pressure.

## Discussion

We show that mouse and human neuroblastoma cells are capable of conversion between two distinct phenotypes dependent on culture conditions, a transition we refer to as *reversible adaptive plasticity*. Identification of several molecular markers of AI and AD phenotypes allowed us to demonstrate that the reversible adaptation occurs during tumor growth *in vivo*. Regardless of which phenotype was used to inoculate mice, the resultant tumors eventually contained cells with a heterogeneous distribution of markers. Importantly, the distinct phenotypes exist in human tumors as well. Chemotherapeutic or growth factor receptor inhibition in mice promoted initial loss of AD or AI heterogeneity, respectively. Simultaneous targeting of both phenotypes with the combination of these drugs gave way to the emergence of other unique phenotypes.

Neuroblastoma is the most common pediatric solid extracranial cancer in which lower-risk tumors are treatable and high-risk disease is frequently fatal. Recent reports indicate the importance of tumor initiating or stem-like cells in tumor resistance and tumor recurrence (Rich, 2007; Wang et al., 2010). Cancer stem cells are described for numerous solid tumors and the unique properties of cancer stem cells suggest a significant role in tumor recurrence following initial effective therapy (Eyler and Rich, 2008; Creighton et al., 2009; Dave and Chang, 2009). The concept of a cancer stem cell has gained much support due to the appeal of a theoretically rare cell with indefinite tumorigenic potential for self-renewal and differential sensitivity to chemotherapeutic agents. In contrast we found that both AI and AD phenotypes were indistinguishable with respect to the self-renewal potential, an undifferentiated state, or expression of stem-like markers (or lack thereof). These findings suggest that both AD and AI phenotypes display stem cell-like characteristics, but we were unable to define a subset of specific tumor stem cells.

The ability of tumor cells to adapt to the environment enables tumors to evade surveillance of the immune system, survive unfavorable conditions, or escape radio- or chemotherapy. This adaptation is most frequently described as a process of selection of the fittest tumor cell clones generated by mutations as a consequence of genetic instability (Marusyk and Polyak, 2010). Thus, under the pressure of the immune response to a particular tumor antigen, preferential outgrowth of tumor variants lacking the specific antigen is frequently found (Zitvogel et al., 2006). This unidirectional selection-based adaptation is one proposed adaptation mechanism whereas EMT is another possible method. During EMT tumor cells undergo a transition to facilitate cell migration (Creighton et al., 2009) and subsequently de-differentiate back into epithelial cells once they have settled in the new location (Thiery and Sleeman, 2006; Creighton et al., 2010). Our present findings indicate that there are additional forms of transitions, since we detected no changes in expression of markers indicative of EMT or MET. It is possible that EMT and the transition we describe (and perhaps other transitions as yet not described, but existing in other tumors) represents the same general process of tumor cell adaptation. This process could be governed by multiple factors including specific molecular programs, tumor origin, tumor location, the abundance/scarcity of nutrients, and/or growth factors and other environmental cues (Magee et al., 2012). We believe that the term “reversible adaptive plasticity” describes multiple possible programs of bidirectional phenotypic conversion. In support of tumor cell conversion is the finding of *in vivo* switching of melanoma cells between so called “proliferative” and “invasive” cancer phenotypes (Hoek et al., 2008). These phenotypes appear different from the ones we describe here since the tumors with proliferative and invasive phenotypes display distinct rates of *in vivo* growth whereas the tumor growth in our *in vivo* model are indistinguishable in growth rate and responsive to environmental cues. Alternatively, visibly different growth rates *in vivo* in melanoma may suggest that melanoma cells cannot transition *in vivo* as readily as neuroblastoma cells do.

Influence of the microenvironment on the plasticity of cancer cells is previously reported (Calabrese et al., 2007; Heddleston et al., 2009; Martinez-Outschoorn et al., 2010; Allen and Louise Jones, 2011). Our findings show adaptation of tumor cells to chemotherapy, as cells displaying AD phenotype displayed greater sensitivity to doxorubicin treatment compared to AI cells. The fact that AD cells proliferate more rapidly than AI cells may explain why doxorubicin was more effective in the former, since doxorubicin inhibits DNA replication of highly proliferative cells (Kotchetkov et al., 2003). The finding that the balance of heterogeneous cell populations present in the tumor *in vivo* is temporarily altered by doxorubicin therapy (with or without metformin) suggests that tumor cell plasticity is a dynamic process that protects tumor cells from sudden unfavorable changes in their environment. The environmental factors and mechanisms that “spontaneously” drive the phenotypic conversion and maintain the cellular heterogeneity in the absence of chemotherapeutic agents are unknown at present. Nevertheless, understanding the mechanism of the present findings should have critical therapeutic implications from pharmacologic and immunologic perspectives and may be an important determinant of tumor prognosis.

In conclusion, the work presented shows that neuroblastoma cells are plastic, dynamic, and respond to their niche. The cells acquire features needed to optimize their ability to survive and the findings described have important implications for targeting tumor cells in transitional states. It is evident that tumor cell sensitivity under specific niches or conditions that favor one phenotype cannot be generalized to predict clinical therapeutic susceptibility. The clinical behavior and successful treatment of neuroblastoma may be dependent on the *reversible adaptive plasticity* described.

## Conflict of Interest Statement

The authors declare that the research was conducted in the absence of any commercial or financial relationships that could be construed as a potential conflict of interest.
